# Predicting response to platinum and non-platinum drugs through bioluminescence resonance energy transfer (BRET) based bio-molecular interactions in platinum resistant epithelial ovarian cancer

**DOI:** 10.1016/j.tranon.2021.101193

**Published:** 2021-08-05

**Authors:** Aniketh Bishnu, Megha Mehrotra, Ajit Dhadve, Shalini Dimri, Abhijit De, Pritha Ray

**Affiliations:** a Imaging Cell Signalling & Therapeutics Lab, Advanced Centre for Treatment, Research and Education in Cancer, TMC, Navi Mumbai 410210, India; b Homi Bhabha National Institute, Anushakti Nagar, Mumbai 400094, India; c Molecular Functional Imaging Lab, Advanced Centre for Treatment, Research and Education in Cancer, TMC, Navi Mumbai 410210, India

**Keywords:** BRET, Chemoresistance, Second line non-platinum drugs, ERK1/2, AKT, HGSOC

## Abstract

Therapy induced rewiring of signalling networks often lead to acquirement of platinum-resistance, thereby necessitating the use of non-platinum agents as second-line treatment particularly for epithelial ovarian cancer (EOC). A prior subject-specific assessment can guide the choice of optimal non-platinum agent/s and possible targeted therapeutic/s. Assessment of protein-protein interactions are superior to simple cytotoxicity assays to determine therapeutic efficacy and associated molecular responses. Utilizing improved PIP3-AKT and ERK1/2 activation Bioluminescence Resonance Energy Transfer (BRET) sensors, we report chemotherapy-induced ERK1/2 activation predominantly in cisplatin-paclitaxel resistant EOC cells and increased activation of both ERK1/2 and AKT in malignant ascites derived cancer cells from platinum-resistant patients but not from treatment-naive or platinum-sensitive relapse patients. Further, majority of the non-platinum drugs except irinotecan increased ERK1/2 activation in platinum-taxol resistant cells as observed by live-cell BRET assessment which were associated with p90RSK1/2 and BAD activation along with upregulation of multidrug transporter gene ABCC1 and cell survival genes like cyclin D1 and Bcl2. Interestingly, only irinotecan was able to sensitize these resistant cells. Altogether, this first report of BRET based sensing of molecular pathway activations in platinum resistant cell lines and patient's derived cancer cells highlight the clinical potential of BRET sensors in management of therapy resistant cancer.

## Highlights

• Dynamic monitoring of therapy induced interactions between target/partners of ERK and AKT kinases in drug resistant ovarian cancer cell utilizing two improved nanoBRET sensors.
• Delineation of the dynamics of ERK1/2 and AKT activation in response to first-line platinum therapy in HGSOC patient derived primary cancer cells of differential platinum resistance status through real-time BRET assay.
• Therapy induced hyper-activated ERK1/2 but not AKT imparts multi-drug resistance against doxorubicin, gemcitabine and etoposide (second line non-platinum agent) in acquired and intrinsically platinum resistant ovarian cancer cells.
• Our study reveals the translational potential of the nanoBRET sensors for predicating efficacy of cytotoxic drugs against platinum-resistant ovarian cancer.

## Introduction

Perturbation in the key signalling hubs due to genetic or extracellular stimuli regulates all aspects of cellular phenotypes and often leads to diseased phenotype like cancer. Chemotherapy, one of the major treatment modalities against cancer often fails to produce significant response due to therapy induced rewiring of signaling networks, which confers cancers cells the ability to evade cytotoxic effects of these therapies. The change in cellular response due to external chemotherapeutic stimuli is governed by differential biomolecular interactions [protein-protein interaction (PPI) or protein-lipid (PLI)], which lead to modulation in key signaling pathways [1,2]. Thus dynamic monitoring of therapy induced modulation through biomolecular interactions is crucial for understanding the molecular basis of chemoresistance and predicting the efficacy of a given therapy. Predicting efficacy of chemotherapeutic drugs on cancer cell lines or cells derived from patient tumours prior to initiation of chemotherapeutic regimen holds a high prognostic significance. Classically, chemotherapeutic drug responses are estimated as a function of reduction in mitochondrial respiration or ATP production which though serves as a gross survival indicator does not predict the underlying molecular alteration responsible for single or multi-drug resistance [3], [4], [5], [6]. Thus understanding these key bio-molecular interactions to estimate the levels of activated signaling pathways may help to determine the choice of 1^st^ /2^nd^/3^rd^ line chemotherapeutics using cancer cell-lines or ex vivo culture of primary cancer cells

Attainment of a highly chemoresistant phenotype renders platinum based chemotherapeutics ineffective in high grade serous ovarian carcinoma (HGSOC). Majority of the patients show disease relapses within 6 months of completion of front-line platinum-taxol therapy and never respond to platinum-again [7]. Currently, there is no effective targeted therapy against platinum-resistant ovarian cancer. Thus, the platinum-resistant cases resort to the available second line chemotherapeutics, which comprise liposomal doxorubicin, topo/irinotecan, gemcitabine and etoposide [8]. The majority of the second line chemotherapeutics show a similar response rate of 15-30% [9,10]. Thus the choice of second line therapy for individual patients relies upon treatment history, cost, availability, associated toxicities and convenience of treatment but rarely on therapy induced molecular responses.

AKT activation driven through PIP3-AKT interaction at plasma membrane and cytoplasmic/nuclear activation of ERK1/2 kinase are the focal points of several signal transduction pathways and reported to impart therapy resistance in various cancers including HGSOC [11], [12], [13], [14]. Previously, we have demonstrated the role of MAPK/ERK1/2 and PI3K/AKT signaling at the onset and maintenance of platinum-taxol resistance in ovarian cancer cells [15], [16], [17]. However, the modulations in these key signaling arms in response to non-platinum agents in chemoresistant ovarian cancer cells were not investigated.

Assessment of AKT and ERK1/2 activation in real-time can be achieved by studying physical interaction of their substrate/partner proteins from live-cells. In recent years, Bioluminescence Resonance energy transfer (BRET), a non-radiative resonance energy transfer based proximity assay, has emerged as a powerful tool for studying PPI, lipid assisted PPI, post-translational modification and protein conformation [18,19]. Briefly, BRET involves distant dependent transfer of energy between a luminescence donor and a florescence acceptor occurred due to interaction between their fusion partner proteins and a higher BRET signal indicates stronger interactions. Earlier, *Renilla* Luciferase (Rluc) based BRET sensors, were utilized to monitor receptor dimerization, GPCR activation, activation of key cellular kinases. However, Nano Luciferase (Nluc) that exhibits high quantum yield and sustained luciferase signal has recently emerged as the choice as donor luciferase. [20]. Such Nluc based BRET platforms were used to monitor STAT3 activation and drug screening in breast cancer cells with high precision [21].

Herein utilizing, Nluc based PIP3/AKT (NAT) and ERK1/2 (NEO) BRET sensors, we reported that ERK1/2 activation but not AKT activation primarily assist in survival of platinum-taxol resistant EOC cells when challenged with same drugs. Intriguingly, platinum induced activation of both ERK1/2 and AKT was only observed in malignant ascites derived cancer cells of platinum-resistant relapse patients but not from therapy-naive or platinum sensitive relapse patients. Further, BRET based monitoring revealed that an increased ERK1/2 activation bestow the platinum-resistant cells a multi-drug resistant phenotype against doxorubicin, gemcitabine and etoposide but not irinotecan as supported by cytotoxicity assay and downstream target activation. Together, our data indicates the unique ability of platinum-resistant cells to activate ERK1/2 in response to platinum and non-platinum drugs to support its survival. To the best of our knowledge, this is the first report on monitoring of chemotherapy induced activation of pro-survival ERK1/2 and AKT arm in cancer cells derived from malignant ascites by BRET technology, which may offer unique application of these sensors in clinical sectors to foresee efficacy of chemotherapeutics.

## Methods

### Cell lines and culture conditions

A2780, OAW42 and SKOV3 were purchased from ATCC and cultured in DMEM, MEM and RPMI respectively (GIBCO, USA), supplemented with 10% fetal bovine serum (Himedia, India) and 1% pencillin-streptomycin (GIBCO,USA) in humidified atmosphere containing 5% CO2 at 37°C. IGF-1 (100nM and 200nM), EGF (200nM), Insulin (250nM and 500nM) were procured Sigma, USA. U0126 (CST, USA), Wortmannin (Sigma, USA) and picropodophyllin (Calbiochem, Germany) treatment were performed at the dosage of 10µM, 200nM and 2µM respectively for 24 hours. Doxorubicin (Sigma, USA), Cisplatin (Sigma, USA), Paclitaxel (Sigma, USA), Etoposide (Etosid^TM^, Cipla), Irinotecan (Irinotel®, Fresenius Kabi) treatment were performed at respective IC50 dosage (Supplementary table 1) for 12/24 hours.

### Development of chemoresistant model

Cisplatin-paclitaxel dual resistant A2780 and OAW42 cellular models were indigenously developed following pulse method of platinum-taxol treatment of sensitive cells for 6 months [15]. Cells were treated with platinum-taxol for 2 hours, following which the drug was removed and cells allowed to recover to 80-90 % confluency. The drug concentration was escalated after every 3 cycles of therapy till acquirement of a highly resistant (∼90% viable at IC_50_ of sensitive sells) phenotype. Cells showing 10 times higher resistance index in comparison to parental counterpart were characterized as late resistant (A2780Dual^LR^/OAW42Dual^LR^) [22].

### Development of PIP3/AKT BRET construct (NAT)

The Nluc-TurboFP_635_ and the Stat3-TurboFP_635_ was kind gifts from Dr. Abhijit De. The PH-AKT-GFP construct was procured from addgene (Plasmid #51465, Tamas Balla lab). The NAT BRET sensor comprises of PH-AKT-Nluc fusion construct (Pleckstrin Homology (PH) domain of AKT fused to nanoluciferase) and the membrane localized TurboFP_635_ protein. The PH-AKT domain was PCR amplified with Nhe-1 and Bgl-II restriction site form PH-AKT-EGFP construct and column purified. The PCR amplified product was then substituted in place of TurboFP_635_ in the TurboFP_635_-Nluc plasmid using the Nhe-I and Bgl-II site to generate PH-AKT-Nluc. Synthetically synthesized oligonucleotides coding for membrane localization signal was annealed and column purified. The membrane localization sequence was derived from N-terminal of neuronal protein GAP3 (20 amino acid) [23]. The annealed oligonucleotide was substituted in place of Stat3 in Stat3-TurboFP_635_ construct using Nhe1 and Sal1 to generate GAP-TurboFP_635_. The BRET pair together was termed NAT.

### Development of BRET sensor for monitoring ERK1/2 activation (NEO)

The Cerulean-EKAR_cytoplasmic_-Venus (Plasmid #18679) and Cerulean-EKAR_nuclear_-Venus (Plasmid #18681) was purchased from Addgene (Karel Svoboda). Briefly, the sensor consists of consensus ERK1/2 target phosphorylation sequence of cdc25 (PRTP), phospho ERK1/2 binding domain (FQFP), a flexible linker of 72 glycine residue and a proline directed phospho binding domain (WW). Binding of phosphorylated ERK1/2 and subsequent CDC25 phosphorylation triggers a conformational change which brings the donor and acceptor proteins in close proximity, thus inducing resonance energy transfer. The Nluc-mOrange construct was a kind gift from Dr. Abhijit De. The full length cytoplasmic and nuclear EKAR was PCR amplified with Bgl-II and Xho-I site and column purified. The PCR amplified product was then introduced in the Nluc-mOrange vector using the Bgl-II and Xho-I site to generate Nluc-EKARcyto/nucl-mOrange (NEO). For construction of Nluc-CEKAR as BRET donor construct for normalization, the CEKAR was PCR amplified and cloned downstream of Nluc by replacing mOrange in the Nluc-mOrange vector using XhoI and BamHI restriction enzyme.

### Site directed mutagenesis

The PH-AKT domain mutant were made in the background of PH-AKT-EGFP and PH-AKT-Nluc by replacing the 14th codon expressing lysine with alanine (K14A_PH-AKT-EGFP/Nluc) by standard site directed mutagenesis (SDM) PCR using the following primers. All constructs were verified by restriction digestion and sequencing. **Forward SDM primer:** ggctgcacGCCcgaggggaatatattaaaacc, **Reverse SDM primer:** cctcggGCgtgcagccagccctcct

### Immunoblotting

Immunoblotting was performed as described previously [21], for evaluating the level of phosphorylated and total ERK1/2, AKT, p90RSK1/2, BAD, mTOR and tubulin. Images were captured in chemidoc (Biorad)

### Cell viability assay

In order to determine IC_50_, cells were incubated with each of the non-platinum drugs at different concentration (Table S1). After 48 hours, cells were incubated with 10% MTT solution for 2 hours followed by addition of 100 µl DMSO. Absorbance was measured at 570nm with background correction at 630nm. Standard thiazolyl blue tetrazolium bromide (MTT, Sigma-Aldrich) method was used to calculate cell viability using the formula: Absorbance (Test)/Absorbance (Control)*100 [24]. The data was fitted into a dose-inhibition curve and plotted.

### Enrichment of tumour cells from ascitic fluid

Patient studies with appropriate consent forms were performed as per Institutional Ethics Guidelines of ACTREC-TMC (IEC#900513). The ascitic fluid collected from chemo naïve, platinum-resistant and platinum-sensitive high grade serous ovarian cancer patients were subjected to centrifugation to obtain cell pellet and treated with RBC lysis buffer to remove contamination of red blood cells. To enrich the tumour cells, depletion of CD90 positive fibroblasts was carried out by Magnetic Assisted cell sorting (MACS, Miltenyi Biotec, USA). CD90 negative fraction was further sorted using FACS for EpCAM (abcam, UK) positive cells. CD90-/EpCAM+ cells thus obtained were cultured in MCDB:M199 (Himedia, India) and experiments were performed within one week of obtaining the cells.

### BRET imaging and analysis

Bioluminescence resonance energy transfer measurements in live-cell populations were performed as previously described [21]. Briefly, cells were transfected with only donor (for normalization) or donor+acceptor plasmids, following which cells were seeded in a 96 black well plate with clear bottom. For BRET imaging, furimazine (1:1000) was added to each well and acquisition was performed at a range of wavelengths from 500-680 nm with 20 nm step increments using IVIS spectrum. For each wavelength at an integration time (10-30 Seconds) was kept constant throughout experiments.

For monitoring, the effect of growth factors on AKT and ERK1/2 activation, cells expressing the respective BRET constructs [NAT, NEO or donor only (PH-AKT-Nluc/Nluc-CEKAR for normalization)] constructs were treated with IGF-1/Insulin/EGF at concentration mentioned in results and BRET signal was acquired at interval of 30 seconds for 20 min at respective donor and acceptor filter. For monitoring the effect specific pharmacological inhibitors on AKT and ERK1/2 activation cells were pre-treated with respective inhibitors for 24 hours followed by IGF-1 stimulation. The donor and acceptor emission was acquired at 500 and 640 nm for NAT and 500 and 560 nm for NEO.

For monitoring the effect of chemotherapeutics on AKT and ERK1/2 activation, cells transfected with either NAT, NEO or donor only (PH-AKT-Nluc/Nluc-CEKAR for normalization) constructs were treated with chemotherapeutics according to the respective IC_50_ of individual cell line (Table S2) for 12 and 24 hours followed by acquisition of donor and emission spectra of NAT and NEO at respective wavelengths. The BRET ratio was calculated by drawing ROI over each well and photon counting in Living image software version 4.5. The corrected BRET ratio represented as milliBRET unit (mBU) was calculated using the formula:

$$mBU=\frac{Avg.\;Rad\left(Acceptor\;filter\right)-Cf*Avg.\;Rad\;\left(Donor\;filter\right)}{Avg.\;Rad\;\left(Donor\;filter\right)}*1000$$

$$Cf=\frac{Avg.\;Rad\left(Acceptor\;filter\right)donor\;only}{Avg.\;Rad\left(donor\;filter\right)donor\;only}$$

### Quantitative real time PCR

Total RNA from A2780Dual^LR^ cells was isolated (Qiagen, Germany) following 12 and 24 hours of doxorubicin or irinotecan treatment and 2 mg of total RNA were converted to cDNA (Thermo Scientific, USA) and qRT-PCR was performed using SYBR-Green (Thermo Scientific, USA) method as described earlier [22]. The relative expression levels of mRNAs were calculated using the formula 2^−ΔCt^ in comparison to GAPDH as an internal control. Primer sequences used: ABCB1: FWD: TGATTGCATTTGGAGGACAA REV: CCAGAAGGCCAGAGCATAAG, ABCC: FWD: TACCTCCTGTGGCTGAATCTG REV: CCGATTGTCTTTGCTCTTCATG, ABCG2: FWD: CTGAGATCCTGAGCCTTTGG REV: AAGCCATTGGTGTTTCCTTG, CYCLIN D1: FWD: TATTGCGCTGCTACCGTTGA REV: CCAATAGCAGCAAACAATGTGAAA, BCL-2: FWD: TCGCCCTGTGGATGACTGA, REV: CAGAGACAGCCAGGAGAAATCA, BAX: FWD: TGGAGCTGCAGAGGATGATTG REV: GAAGTTGCCGTCAGAAAACATG, GAPDH: FWD: TGCACCACCAACTGCTTAGC REV: GGCATGGACTGTGGTCATGAG

### Statistical analysis

All the data has been represented as mean ± SEM (n>=3). Student's t-Test was applied to evaluate the significance of the changes observed. A p-value < 0.05 was considered statistically significant.

## Results

### PH-AKT-Nluc/TurboFP_635_-mem (NAT) BRET sensor accurately predicts PIP3/AKT activation

**Fig. 1:**
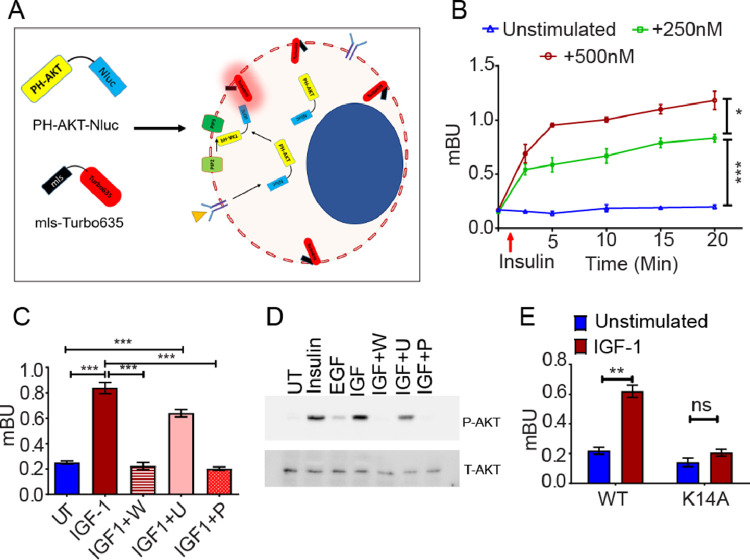
**Monitoring of PIP3/AKT activation dynamics** (A) Schematic representation of the working principle of NAT biosensor. (B) Graph representing dose and time dependent increase in BRET ratio (mBU) post insulin treatment in A2780 cells. (C) Wortmannin (W, 200nM, 0.26-fold) and picropodophyllin (P, 2mM, 0.24-fold) but not U0126 (U, 10µM, 0.75-fold) specifically reduced IGF-1 (200nM) induced NAT BRET ratio in A2780 cells (D) Immunoblot depicting increased AKT phosphorylation post treatment with IGF-1, EGF and insulin, while pretreatment of cells with wortmannin or picropodophyllin significantly reduces IGF-1 induced AKT phosphorylation in A2780 cells (E) Site directed mutagenesis in the PH domain (K14A) of AKT significantly abrogates IGF-1 induced PH-AKT PIP3 interaction. (Data represents mean ± SEM of at least two independent experiments, ns indicates non-significant, * indicates p < 0.05, ** p < 0.005, ***p < 0.0005 as calculated by unpaired t-test).

Membrane translocation of AKT via its Pleckstrin Homology (PH) domain upon conversion of PIP2 to PIP3 by phosphoinositol-3-kinase is a crucial and initial step of AKT activation (Fig 1A). To capture this step, the PH domain of AKT was fused to nanoluciferase (donor) and membrane targeted TurboFP_635_ (acceptor) were utilized to construct the NAT BRET sensor. PH-AKT-Nluc yielded 127 fold higher luciferase signal compared to PH-AKT-Rluc and did not suffer proteolytic cleavage when transiently transfected in A2780 cells (Fig S1A-B). Efficient energy transfer between NLuc and TurboFP_635_ at the characteristic emission maxima (NLuc: 500 nm and TurboFP_635_: 640 nm) with a BRET ratio of 231.94 mBu was observed upon ectopic expression of TurboFP_635_-NanoLuc in A2780 cells (FigS1C). Since geometric orientation of fusion protein can affect resonance energy transfer, Nluc was fused to PH-AKT at both orientation. Nluc fused to the C-terminus of PH-AKT (PH-AKT-Nluc) displayed higher BRET ratio compared to N-terminal orientation post insulin treatment, indicating optimal energy transfer in C-terminal geometric orientation (FigS1D). A dose-dependent increase in AKT activation was observed post insulin treatment in A2780 and MCF7 cells transiently transfected with NLuc-PH-AKT & TurboFP_635_-mem reporters (Fig 1B and S1E). Similarly IGF-1 induced an equivalent increase in BRET ratio post 15 min of stimulation but a comparatively lower BRET ratio was observed post EGF treatment in these cells indicating the sensitivity of the system (FigS1F). Pre-treatment of cells with wortmannin (PI3KCA inhibitor) and picropodophyllin (IGF1R inhibitor) prevented IGF-1 induced AKT activation by 0.26 fold and 0.24 fold respectively, while U0126 (MEK1/2 inhibitor) pre-treatment minimally (0.75 fold) inhibited IGF-1 induced AKT activation thus indicating the specificity of NAT sensor (Fig 1C). The BRET data was corroborated by similar modulation in phospho AKT level post treatment with different growth factors and inhibitors by immunoblotting (Fig 1D). Interestingly, introduction of a point mutation (K14A) in the PH domain of AKT (Nluc-PHK14A-AKT) completely abolished PIP3 PH-AKT interaction and abrogated IGF-1 induced increased BRET signal (Fig 1E and S1G).

### Nluc-EKAR-mOrange (NEO) BRET sensor accurately predicts ERK1/2 activation

**Fig. 2:**
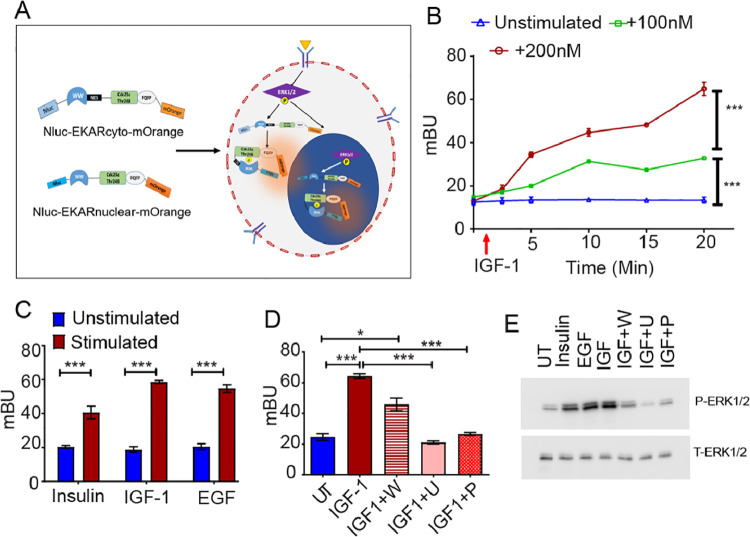
**Monitoring of ERK1/2 activation dynamics** (A) Schematic representation of the working principle of NEO biosensor. (B) Graph representing dose and time dependent increase in BRET ratio (mBU) post IGF-1 treatment in A2780 cells. (C) IGF-1 (200nM) and EGF (200nM) induced highest ERK1/2 activation post 15 min of stimulation in A2780 cells (D) U0126 (U, 10µM, 0.32-fold) and picropodophyllin (P, 2mM, 0.40-fold) but not wortmannin (W, 200nM, 0.70-fold) specifically reduced IGF-1 (200nM) induced NEO BRET ratio in A2780 cells (E) Immunoblot depicting increased ERK1/2 phosphorylation post treatment with IGF-1, EGF and insulin, while pretreatment of cells with U0126 or picropodophyllin significantly reduces IGF-1 induced ERK1/2 phosphorylation (Data represents mean ± SEM of at least two independent experiments, ns indicates non-significant, * indicates p < 0.05, ** p < 0.005, ***p < 0.0005 as calculated by unpaired t-test).

Along with NAT, a BRET based ERK1/2 activity reporter (NEO) was developed by replacing the fluorophores of an existing FRET based Extracellular signal-regulated Kinase Activity Reporter (EKAR_cytoplasmic_ and EKAR_nuclear_) with NanoLuc and mOrange (Nluc-EKAR-mOrange) (details are mentioned in method section, Fig 2A)(23). Efficient energy transfer (257.94) between the Nluc-mOrange BRET pair and stability of the fusion protein was validated in A2780 cells (FigS2A, S1B). IGF-1 stimulation for 15 min induced higher BRET ratio in A2780 cells transfected with Nluc-EKAR-mOrange (3.1-fold) compared to that of mOrange-EKAR-Nluc (2.2-fold) indicating the optimal orientation of the BRET sensor (FigS2B). To monitor the total ERK1/2 activity (Cytoplasmic+Nuclear) we transfected both the constructs together in all the experiments. Similar to AKT BRET sensor, a dose and time dependent increase in NEO BRET ratio was observed in A2780 and MCF7 cells post IGF-1 treatment (Fig 2B and S2-C). Both IGF-1 and EGF induced higher BRET ratio compared to insulin in A2780 cells (Fig 2C). Pre-treatment of A2780 cells with U0126 or picropodophyllin significantly abolished IGF-1 induced BRET ratio (0.40-fold) while wortmannin partially prevented (0.70-fold) the same indicating specificity of the system (Fig 2D). A similar trend in ERK1/2 phosphorylation was observed post treatment with different growth factors and inhibitors by immunoblotting (Fig 2E).

### Dynamic modulation of ERK1/2 and AKT activation in sensitive and chemoresistant cell lines and ascites derived primary cancer cells

**Fig. 3:**
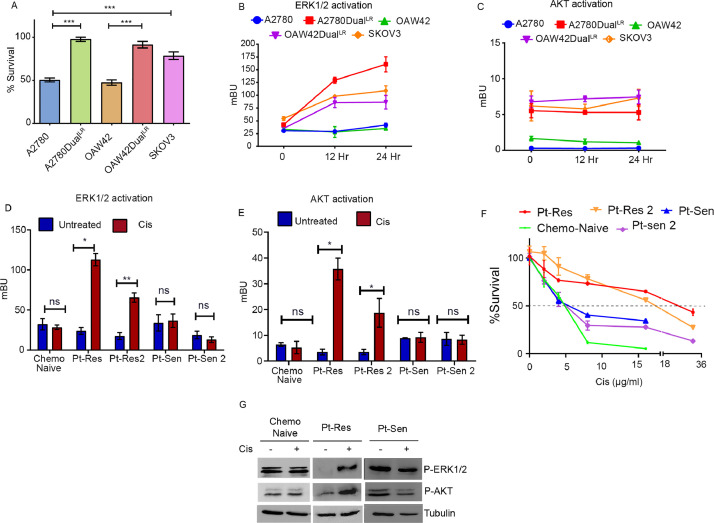
**Therapy induced modulation in ERK1/2 and AKT predicts response to platinum** (A) Graph representing reduced sensitivity of platinum against A2780Dual^LR^, OAW42Dual^LR^ and SKOV3 cells towards cisplatin. (B) Graph representing significantly increased NEO BRET ratio in A2780Dual^LR^, OAW42Dual^LR^ and SKOV3 cells post 12 and 24 hours of platinum treatment. (C) Platinum treatment did not significantly alter NAT BRET ratio in both sensitive (A2780 and OAW42) and resistant cells (SKOV3 and A2780 Dual^LR^ /OAW42Dual^LR^). The basal NAT BRET ratio was higher in resistant cells compared to sensitive cells. (D-E) Live-cell quantification of ERK1/2 and AKT activation in patient derived malignant cells expressing NAT and NEO BRET sensor; increased NAT and NEO BRET ratio was observed post platinum treatment specifically in cells derived from both platinum-resistant relapse patients (Pt-Res and Pt-Res2) but not in chemo-naïve or platinum sensitive relapse (Pt-Sen and Pt-Sen 2) patients. (F) Graph indicating increased platinum tolerance specifically in platinum-resistant relapse case (Pt-Res: 20µg/ml, Pt-Res 2: 16 µg/ml) compared to platinum-sensitive relapse (Pt-Sen: 4 µg/ml, Pt-Sen 2: 5µg/ml) and Chemo-naïve (4µg/ml) patients as observed by MTT assay. (G) Immunoblot depicting increased platinum induced ERK1/2 and AKT phosphorylation specifically in cells derived from platinum-resistant relapse patients. (Data represents mean ± SEM of at least two independent experiments, ns indicates non-significant, * indicates p < 0.05, ** p < 0.005, ***p < 0.0005 as calculated by unpaired t-test).

Previously, we described the development of dynamic models of platinum-taxol dual resistance following pulse method of drug treatment in A2780 and OAW42 EOC cells and categorized them as early (A2780Dual^ER^/OAW42Dual^ER^) and late (A2780Dual^LR^/OAW42Dual^LR^) based on their resistant indices [16,17]. Combinatorial treatment of platinum and taxol induced only 2.2% and 8.5% cell death in A2780Dual^LR^ and OAW42Dual^LR^ cells in comparison to parental A2780 (49.5%) and OAW42 (52.5%) cells. A similar treatment induced 22.3% cell death in SKOV3, an intrinsically platinum resistant cell line (Fig 3A). Dual^LR^ of both models show enhanced AKT activation compared to their sensitive counterparts while SKOV3 cells possess high levels of both activated ERK1/2 and AKT (FigS3A).

Interestingly, platinum-taxol treatment gradually increased NEO BRET signal in A2780Dual^LR^, OAW42Dual^LR^ and SKOV3 cells at 12 and 24 hours compared to A2780 and OAW42 cells indicating a drug induced dynamic interaction of ERK1/2 target with ERK1/2. Contrarily, basal NAT BRET ratio was already high in SKOV3 and Dual^LR^ cells of both models compared to sensitive A2780 and OAW42 cells, and did not increase further post cisplatin-paclitaxel treatment (Fig 3B-C) indicating a saturation in AKT partner interaction with AKT at the membrane. A similar increase in phospho-ERK1/2 but not phospho-AKT level was observed in both acquired and intrinsically resistant ovarian cancer cells post cisplatin-paclitaxel treatment at the protein level (FigS3B).

Next, we monitored therapy induced modulation of ERK1/2 and AKT activation in CD90^−ve^EPCAM^+ve^ ovarian cancer cells isolated from malignant ascites of HGSOC patients with differential drug resistant status. The epithelial nature of these cells were confirmed by CK18 staining (data not shown). Interestingly, increased NEO (4.72- and 3.76-fold) and NAT BRET (10.11- and 5.30-fold) ratio were specifically observed in live-cells derived from platinum-resistant patients respectively treated with platinum. Such changes in BRET ratio were not observed in cells derived from chemo-naive and platinum-sensitive relapse patients (Fig 3D-E). An increased phospho-ERK1/2 and AKT level was observed specifically in cells obtained from platinum-resistant patients post platinum challenge (Fig 3G). Cells derived from both the platinum-resistant patients also showed higher platinum tolerance (20 and 16µg/ml respectively) in comparison to chemo-naive (4.5 µg/ml) and both the platinum-sensitive (4 and 4.5 µg/ml respectively) patients (Fig 3F).

### ERK1/2 activation predicts response to non-platinum drugs in platinum-taxol resistant ovarian cancer cells

**Fig. 4:**
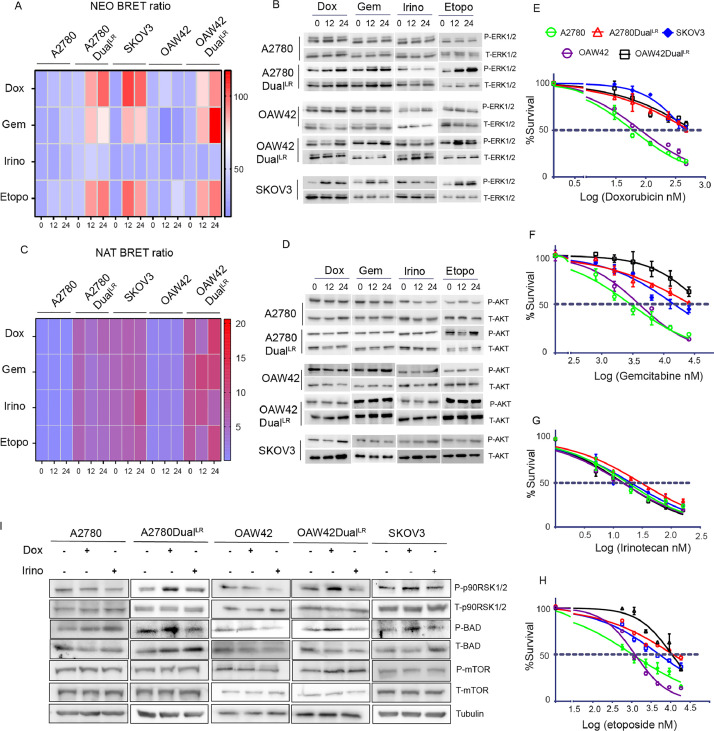
**Therapy induced ERK1/2 activation alleviates cytotoxicity of second line non-platinum agents in platinum-taxol resistant ovarian cancer cells:** (A, C) Heat map representing alteration in NEO and NAT BRET signal, respectively, in sensitive (A2780 and OAW42) and therapy resistant (A2780Dual^LR^, OAW42Dual^LR^ and SKOV3) cells post treatment with IC_50_ dosage (TableS2) of doxorubicin (Dox), gemcitabine (Gem), irinotecan (Irino) and etoposide (Etopo). A significant increase in NEO BRET signal was observed in therapy resistant cells post treatment with doxorubicin, gemcitabine, etoposide but not irinotecan (B, D) Immunoblot depicting increased ERK1/2 phosphorylation but not AKT phosphorylation specifically in therapy resistant cells treated with majority of the non-platinum agents except irinotecan. (E-H) Graph representing percent survival versus log transformed concentration of non-platinum agents in sensitive (A2780 and OAW42) and therapy resistant (A2780Dual^LR^, OAW42Dual^LR^ and SKOV3) cells post 72 hours of treatment. Interestingly, only irinotecan sensitized platinum resistant cells to therapy. (Concentration range is represented in supplementary TableS1) (I) Immunoblot depicting increased phospho p90RSK1/2 and phospho BAD level specifically in A2780Dual^LR^, OAW42Dual^LR^ and SKOV3 cells post doxorubicin treatment compared to irinotecan. (Data represents mean ± SEM of at least two independent experiments, ns indicates non-significant, * indicates p < 0.05, ** p < 0.005, ***p < 0.0005 as calculated by unpaired t-test).

Since our sensors accurately predicted platinum induced AKT or ERK1/2 activation through interactions of their target/partner in platinum resistant EOC cells, we were interested to understand their potential for non-platinum drugs (doxorubicin, gemcitabine, etoposide and irinotecan). Doxorubicin treatment (24 hours) increased NEO BRET signal in A2780Dual^LR^ (2.3-fold), OAW42Dual^LR^ (3.5-fold) and SKOV3 (2.6-fold) cells, while no significant change was observed in A2780 and OAW42 cells. Increased NEO BRET signal was also observed post 24 hours of gemcitabine and etoposide treatment in specifically A2780Dual^LR^ (1.96- and 2.22-fold), OAW42Dual^LR^ (3.35- and 2.70-fold) and SKOV3 (2.7- and 3.11-fold) cells but not in A2780 and OAW42 cells. Intriguingly, Irinotecan treatment did not result any change in NEO BRET ratio in chemoresistant or chemosensitive cells (Fig 4A). Contrarily, NAT BRET ratio did not alter significantly post treatment with any of the non-platinum drug in both chemoresistant and sensitive cells (Fig 4C). Immunoblotting revealed a similar increase in phospho-ERK1/2 level post doxorubicin, gemcitabine and etoposide treatment but not irinotecan treatment while phospho-AKT level remained unaltered post treatment with any of these drugs in both resistant and sensitive cells (Fig 4B, D). Intriguingly, A2780Dual^LR^, OAW42Dual^LR^ and SKOV3 cells showed increased tolerance (Resistance index [RI]: 8.9, 7.70 and 7.45 respectively) to doxorubicin in comparison to A2780 and OAW42 cells. Similarly, A2780Dual^LR^ (RI: 9.41 and 10.94), OAW42Dual^LR^ (RI: 10.34 and 8.86) and SKOV3 (RI: 5.01 and 4.86) cells also showed resistance to gemcitabine and etoposide respectively. However, irinotecan treatment induced similar levels of cytotoxicity in both sensitive and resistant cells (Fig 4E-H).

### ERK1/2 promotes survival and anti-apoptotic signature in platinum-taxol resistant cells

**Fig. 5:**
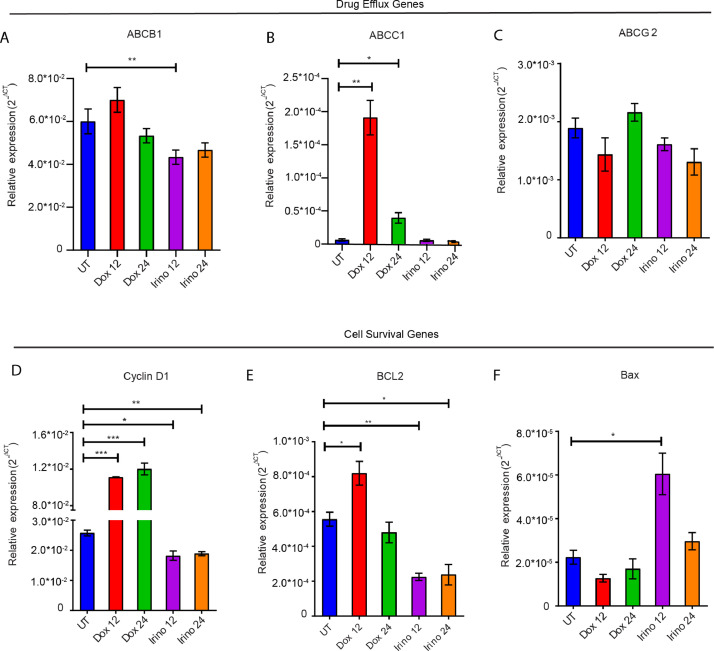
**Modulation in drug-efflux and cell survival genes in response to non-platinum agents in platinum-taxol resistant cells:** (A-C) Graph representing relative expression of Drug efflux genes post 12 and 24 hour treatment of doxorubicin and irinotecan in A2780Dual^LR^ cells. A significant increase in ABCC1 (30.6- and 6.3-fold) expression was observed post doxorubicin treatment at 12 (Doxo12) and 24 (Doxo24) hours respectively, while irinotecan treatment did not alter the expression of ABCB1, ABCC1 and ABCG2 expression significantly. (D-F) Graph representing relative expression of cell survival genes post 12 and 24 hour treatment of doxorubicin and irinotecan in A2780Dual^LR^ cells. A significant increase in Cyclin D1(4.3- and 4.6-fold) and Bcl-2 (1.47- and 0.9-fold) expression was observed post doxorubicin treatment at 12 (Doxo12) and 24 (Doxo24) hours respectively, while irinotecan treatment reduced expression of Cyclin D1 (0.70- and 0.73-fold) and Bcl-2 (0.40- and 0.42-fold) at 12 (Irino12) and 24(Irino24) hours respectively and with concomitant increase in pro-apoptotic Bax (2.73- and 1.32-fold) expression at 12 and 24 hours respectively. Gapdh expression was utilized as internal control (Only statistically significant changes are depicted in the graph with Asterisk, Data represents mean ± SEM of two independent experiments, * indicates p < 0.05, ** p < 0.005, ***p < 0.0005 as calculated by unpaired t-test).

Increased p90RSK1/2 phosphorylation, a downstream target of ERK1/2, was observed in Dual^LR^ and SKOV3 cells post doxorubicin treatment while irinotecan treatment did not alter p90RSK1/2 phosphorylation. Increased inhibitory phosphorylation of pro-apoptotic protein BAD at serine 112 residue was also observed specifically in platinum-taxol resistant and SKOV3 cells post doxorubicin but not after irinotecan challenge. No alteration in mTORC1 level, a bonafide AKT target, was observed post doxorubicin or Irinotecan treatment in sensitive or chemoresistant cells (Fig 4I). A significantly increased expressions of multidrug transporter gene ABCC1 and cell survival genes like cyclin D1 were observed post 12 and 24 hours of doxorubicin treatment in A2780Dual^LR^ cells. Increased expression of Bcl-2 was also observed post doxorubicin treatment specifically at 12 hours while irinotecan treatment reduced expressions of ABCC1, cyclinD1 and Bcl-2 along with increased Bax expression (pro-apoptotic) (Fig. 5A-F).

## Discussion

Acquirement of platinum resistance is a major obstacle in management of HGSOC as significant number of the cases show disease relapse within a very short period (6-12 months) and do not respond to platinum agents anymore. Several efforts are already underway to target key molecular alterations and pathways for this hapless group. However, it's also important to evaluate and optimize the currently available therapeutic options for management of platinum-resistant disease. Monitoring of the dynamic modulations of bio-molecular interactions in response to various drugs is critical to understand the underlying events that assist in cancer cell survival specifically in the drug resistant ones. BRET is a highly sensitive technique to monitor bio-molecular interactions in real-time from live-cell to living subjects. Herein, using two improved BRET sensors (NAT-BRET (AKT activation sensor) and NEO-BRET (ERK1/2 activation sensor)), we showed that the principal surviving mode of platinum-taxol resistant EOC cells involved gradual ERK1/2 activation while highly activated AKT level remained unchanged after drug treatment. Intriguingly, both NAT-BRET and NEO-BRET biosensors showed in-creased BRET signal in primary cancer cells derived from platinum-resistant relapse patients but not from treatment-naive or platinum-sensitive relapse patients. Further, we showed that NEO-BRET system was able to distinguish between the responses to non-platinum drugs in multiple platinum resistant cell lines and demonstrated that activated ERK1/2 and its down-stream targets play a major role in conferring resistance to multiple drugs. Irintotecan, but not doxorubicin or gemcitabine or etoposide was able to induce cell death and did not enhance NEO-BRET ratio in platinum resistant cell lines. Thus these improved BRET sensors have the potential to determine efficacy of drugs in a high-throughput platform and to tailor response for individual patients based on their state of the disease.

Alteration in PPI network can lead to various diseases and also contribute to the aberrant activation of cell survival pathways leading to cancer progression and therapy resistance. Among the several methods to study PPI, BRET based assay systems has emerged as powerful tool to monitor receptor dimerization, conformation and activation of G-protein coupled receptors, receptor ligand interaction, etc. [25]. The RLuc-PH-AKT/YFP-mem BRET sensor was reported to accurately monitor ligand induced PIP3 formation and AKT activation in MCF7 cells and used as prognostic tool to measure PI3K activity in human serum [26]. Similarly, Rluc-EKAR-Venus (REV), a BRET based ERK1/2 activity reporter was utilized to dynamically monitor ERK1/2 activation in primary cultures of hippocampal neurons isolated from 17 day embryonic mice in response to growth factors and pharmacological inhibitors [26,27]. Though both systems worked suitably on their own, the choice of donor luciferase has been shifted to nanoluciferase in recent years due to its superior brightness and quantum yield. Utilizing Nluc, a phospho BRET platform has recently been developed to monitor canonical as well as non-canonical STAT3 activation in breast cancer cells [28]. Herein, by combining brighter and commercially available NLuc donor with either mOrange or TurboFP_635_ acceptors, NEO (ERK1/2 activation sensor) and NAT (AKT activation sensor) BRET systems were developed respectively. Incorporation of Nluc offered increased quantum yield and spectral resolution when paired with mOrange (100 nm) or TurboFP_635_ (180nm) compared to Rluc-YFP/Rluc8-Venus (∼55nm), which significantly increased signal/background ratio and sensitivity of the systems.

The extent (level of phosphorylated form) and duration of the phosphorylated status are the net results of dynamic and transient interactions between several downstream molecules of a respective RTK-ligand interaction [29]. Thus the level of ERK1/2 and AKT activation depends on the type of RTK-ligand interaction and their expression level. Our NAT and NEO BRET sensors precisely portrayed different ligands (IGF-1, Insulin or EGF) induced differential activation of ERK1/2 and PIP3-AKT in A2780 and MCF7 cell lines with maximal activation caused by IGF-1. Such increased level of AKT activation was earlier reported in MCF7 cells stimulated with IGF-1 compared to EGF stimulation [26]. Presence of specific kinase inhibitors (wortmannin and U0126), RTK inhibitor (PPP) and inactivating mutations (K14A-PH-AKT) reversed the IGF-1 induced increase in NAT and NEO BRET ratio, indicating the specificity of the sensors in dynamic monitoring of these key signalling events.

PIP3/AKT and ERK1/2 are the key signalling hubs, reported to play a crucial role in development of therapy resistance in multiple carcinoma including ovarian cancer [11,14,[30], [31], [32]]. Previously we demonstrated the role of these pathways during initiation and maintenance of chemoresistance in two indigenously developed acquired chemoresistant cellular models of ovarian carcinoma [15,33,34]. Re-challenging platinum-taxol resistant Dual^LR^ cells and intrinsically platinum-resistant SKOV3 cells with cisplatin-paclitaxel specifically increased NEO BRET ratio and ERK1/2 phosphorylation without any significant alteration in the NAT-BRET ratio or AKT phosphorylation, implying prominent role of ERK1/2 mediated signaling in platinum-taxol induced therapeutic stress. Such trend was also reported by multiple studies encompassing hepatocellular as well as ovarian carcinoma [35,36]. However, both ERK1/2 and AKT activations were captured by NEO & NAT-BRET systems and by western blots in malignant ascites derived cancel cells from platinum resistant HGSOC patients. Such trends were not observed from cancer cells derived from chemo-naïve or platinum-sensitive relapse patients. To the best of our knowledge, this is the first report of capturing dynamic activation status of kinases through BRET based protein-protein interactions in primary cancer cells of differential drug resistance status which is far superior than currently practiced RPPA or IHC based methods. Our data confirms that platinum induced ERK1/2 activation is a molecular signature for platinum-resistance and probably bestows survival advantage for these cells. However, discrepancy in NAT BRET ratio enhancement between cell lines and primary cancer cells might arise from heterogeneous nature of these cells or from inherent genetic alterations in PI3K-AKT pathway. In parallel to our observation, a gene microarray study of 28 patients with HGSOC demonstrated that samples relatively resistant to platinum chemotherapy showed enrichment of genes involving PI3K and ERK1/2 signaling when compared to platinum-sensitive tumors [37]. Thus the role of activated AKT in imparting platinum resistance cannot be ruled out and need to be investigated in detail.

Currently available therapeutics for platinum-resistant HGSOC include Doxorubicin, Gemcitabine, Irinotecan (preferred over topotecan due to reduced toxicity) and etoposide. These drugs show a similar response rates (10-15%), PFS (3-4 months), and OS (∼12 months) against platinum-resistant HGSOC [8,9]. The efficacy of these non-platinum agents reduce further upon attainment of multidrug resistance phenotype by the cancer cells [38]. Development of resistance to doxorubicin and partially to topotecan had been reported in paclitaxel resistant ovarian cancer cells [39]. Thus, prior assessment of the efficacy of a chosen second line drug for a particular patient can be of great benefit. Classically, MTT or other cytotoxicity based assays are utilized to monitor efficacy of chemotherapeutics drugs against cancer cells which however fail to monitor the underlying molecular response. We observed increased NEO BRET ratio and phospho-ERK1/2 levels, indicative of ERK1/2 activation specifically in chemoresistant cells post treatment with doxorubicin, gemcitabine and etoposide but not of irinotecan. On the other hand NAT-BRET ratio and phospho-AKT levels did not alter post treatment. We also observed that cisplatin-paclitaxel dual resistant A2780/OAW42Dual^LR^ and SKOV3 cells were cross-resistant to doxorubicin, gemcitabine and etoposide. Intriguingly, these resistant cells failed to activate ERK1/2 in response to irinotecan and also showed significantly higher sensitivity towards irinotecan. Thus our study firmly establishes that the ERK1/2 activation as a regulator of multi-drug resistance and as a predictor of poor therapeutic response. The therapy induced increased ERK1/2 activation was found to promote an anti-apoptotic, pro-survival cellular phenotype via p90^RSK1/2^-BAD signalling while irinotecan treatment did not alter the level of p90^RSK1/2^ and BAD phosphorylation. Interestingly, an increased ERK1/2 activation post doxorubicin treatment was also found to foster both drug efflux and cell survival by upregulation of ABCC1, Cyclin D1 and Bcl-2 expression while irinotecan treatment reduced expression of MDR, cell survival genes and promoted expression of pro-apoptotic Bax gene. Activation of ERK1/2 was reported to promote multi-drug resistant phenotype in other cancers as well [40], [41], [42]. Combinatorial treatment of U0126 and adriamycin was reported to sensitized multidrug resistant HEPG2 cells [43]. Increased ERK1/2 activation post gemcitabine treatment in gemcitabine resistant pancreatic cancer cells, Bxpc-3, was reported to promote MDR phenotype, which reversed upon combinatorial treatment of gefitinib and gemcitabine [42]. Thus, pharmacological targeting of ERK1/2 activation or application of chemotherapeutics that do not promote ERK1/2 activation may significantly improve the therapeutic outcome in platinum-resistant HGSOC. These NAT and NEO BRET platforms are thus amenable for high throughput testing of multiple drugs prospectively as well as for prediction of possible targeted therapy in a personalized manner.

## Declaration of Competing Interest

The authors declare no competing financial interest.

## Appendix Supplementary materials

Image, application 1
