# Supplementary material for: Predicting response to platinum and non-platinum drugs through bioluminescence resonance energy transfer (BRET) based bio-molecular interactions in platinum resistant epithelial ovarian cancer
Source: Transl Oncol. 2021 Aug 5;14(11):101193. doi: 10.1016/j.tranon.2021.101193 (PMC8353342; doi:10.1016/j.tranon.2021.101193)
Supplement: Supplementary file 1 [file mmc1.docx]

Supplementary figure 1


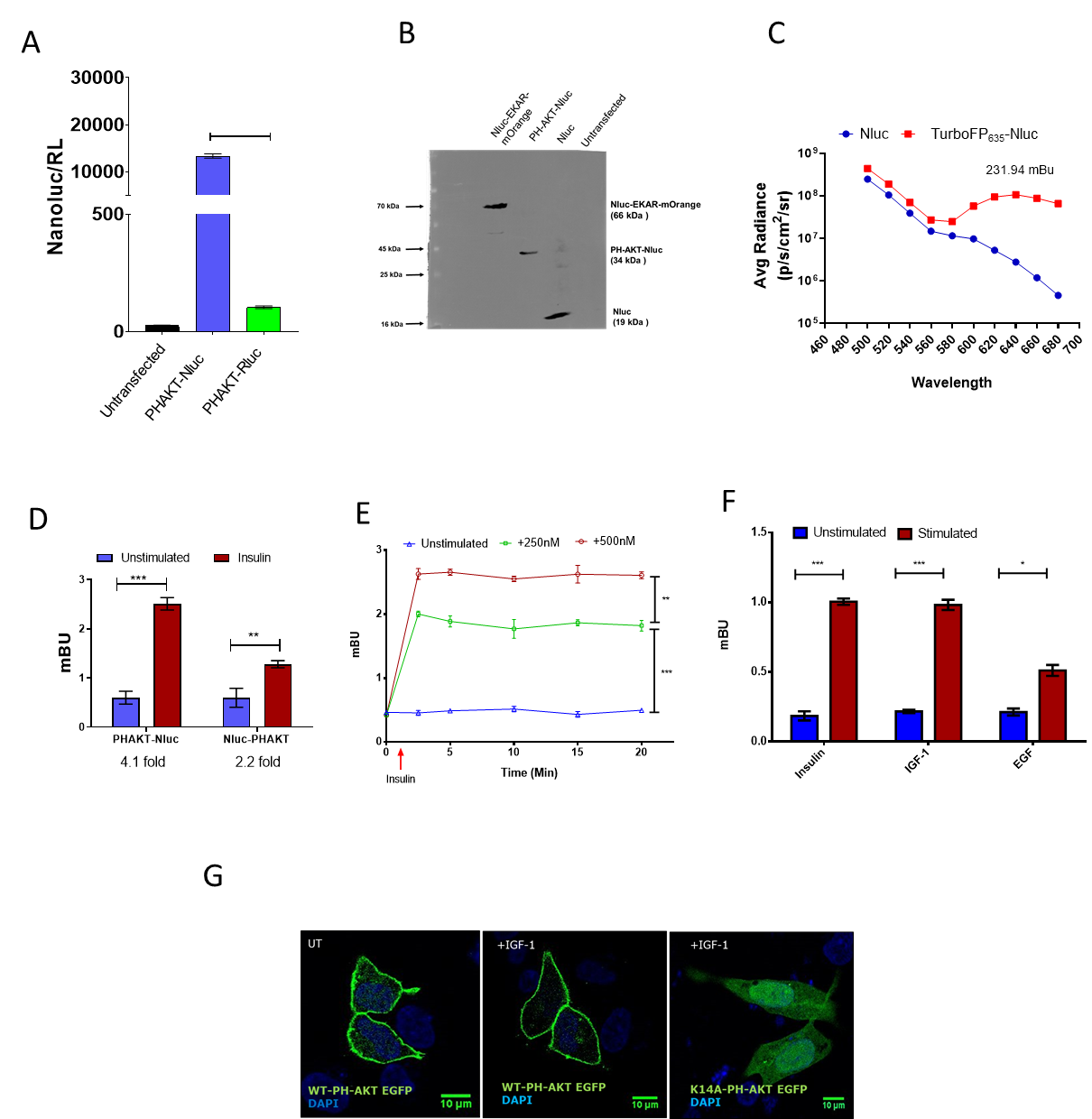


**Supplementary figure 1: Validation of NAT BRET sensor**

(A) Graph representing 124-fold increase in luciferase activity in A2780 cells expressing PH-AKT-Nluc (PH-AKT-Nluc) in comparison to cells expressing PH-AKT-Rluc (B) Immunoblot depicting increased molecular weight of PH-AKT-NLuc fusion protein (34 kDa) and nanoluciferase-EKAR-mOrange (66 KDa) fusion protein in comparison to nanoluciferase (Nluc, 19 kDa). (C) Spectral scan of nanoluciferase (Nluc) and TurboFP_635_-Nluc fusion protein indicating resonance energy transfer (231.9 milliBRET unit, mBu) between Nluc and TurboFP_635_ at 640 nm in A2780 cells (D) Fusion of Nluc in the C-terminal of AKT show increased efficiency of resonance energy transfer in comparison to Nluc fused in the N-terminal of AKT when paired with membrane localized TurboFP635 in A2780 cells (E) Dose and time dependent increase in NAT BRET ratio post insulin (250nM and 500nM) treatment in MCF7 cells (F) Increased NAT BRET ratio in A2780 cells post treatment (15 minute) with Insulin (5.5-fold), IGF-1 (4.6-fold) and EGF (2.42-fold) (G) Representative confocal images depicting membrane translocation of wild type PHAKT (WT-PH-AKT-EGFP) post IGF-1 treatment which gets impaired severely in K14A point mutated PH-AKT (K14A-PH-AKT-EGFP) (Scale 10µm).

Supplementary figure 2


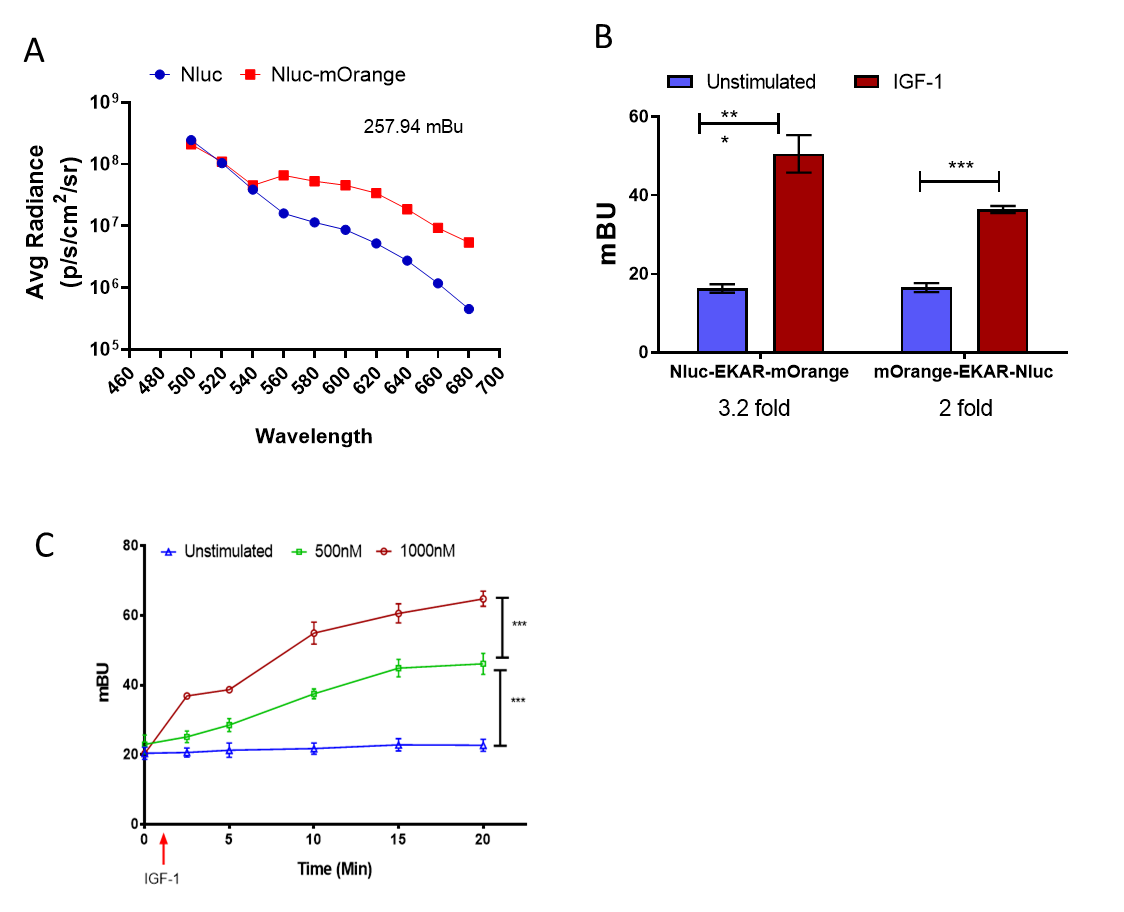


**Supplementary figure 2: Validation of NEO BRET sensor**

(A) Spectral scan of nanoluciferase (Nluc) and nanoluciferase-mOrange (Nluc-mOrange) fusion protein indicating resonance energy transfer (257.9 milliBRET unit, mBu) between Nluc and mOrange at 560 nm in A2780 cells (B) Fusion of Nluc in the N-terminal of EKAR show increased efficiency of resonance energy transfer in comparison to Nluc fused in the C-terminal of EKAR in A2780 cells (C) Dose and time dependent increase in NEO BRET ratio post IGF-1 (500nM and 1000nM) treatment in MCF7 cells.


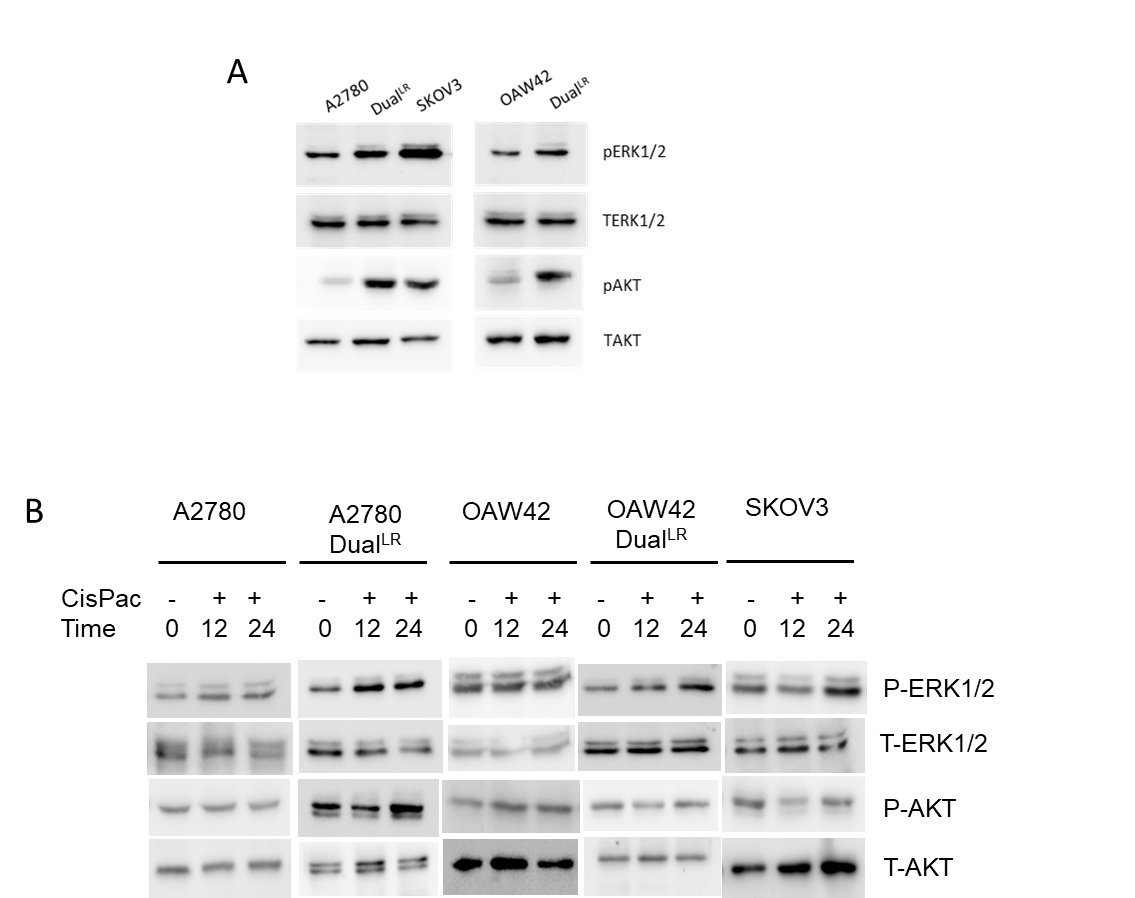
Supplementary figure 3

**Supplementary figure 3: Basal and platinum-taxol induced ERK1/2 and AKT activation**

(A) Immunoblot depicting increased basal activation of AKT and ERK1/2 in cisplatin-paclitaxel dual resistant A2780Dual^LR^, OAW42Dual^LR^ cells and intrinsically platinum resistant SKOV3 cells in comparison to sensitive A2780 and OAW42 cells (B) Immunoblot depicting increased platinum-taxol induced increased ERK1/2 phosphorylation specifically in cisplatin-paclitaxel dual resistant A2780Dual^LR^, OAW42Dual^LR^ cells and intrinsically platinum resistant SKOV3 cells in comparison to sensitive A2780 and OAW42 cells while the level of AKT phosphorylation did not alter significantly.

Supplementary Table 1

Concentrations of respective chemotherapeutic drug used for MTT assay

| **Drugs** | **Concentration range** |
| --- | --- |
| Doxorubicin | (0-480 nM) |
| Gemcitabine | (0-25.6 µM) |
| Irinotecan | (0-160 nM) |
| Etoposide | (0-18.4 µM) |

Supplementary Table 2

IC_50_ concentration of chemotherapeutic drugs

| **Cell line** | **Concentration** | | | | |
| --- | --- | --- | --- | --- | --- |
|  | Cisplatin-paclitaxel | Doxorubicin | Gemcitabine | Irinotecan | Etoposide |
| A2780 | 50 ng/ml+8.5 ng/ml | 60 nM | 2.9 µM | 17.9 nM | 1.4 µM |
| OAW42 | 72 ng/ml+14 ng/ml | 82 nM | 4 µM | 14.6 nM | 1.2 µM |
| SKOV3 | 300 ng/ml+30 ng/ml | 454 nM | 18.9 µM | 20.46 nM | 9.3 µM |
| A280Dual^LR^ | 500 ng/ml+85 ng/ml | 540 nM | 27.5 µM | 32.5 nM | 11.53 µM |
| OAW42Dual^LR^ | 720 ng/ml+ 140 ng/ml | 548 nM | 41.7 µM | 14.9 nM | 10.7 µM |
